# 311. Defining the magnitude of desirability of outcome ranking (DOOR) differences in effectiveness of Ceftazidime-Avibactam (CZA) versus Ceftolozane-Tazobactam (C/T) for multidrug-resistant (MDR) *Pseudomonas aeruginosa* infections in the United States (CACTUS)

**DOI:** 10.1093/ofid/ofae631.101

**Published:** 2025-01-29

**Authors:** jason M Pogue, Lilian M Abbo, Renee Ackley, Samuel L Aitken, Benjamin Albrecht, Ahmed Babiker, Rachel Burgoon, Kimberly C Claeys, Brooke Curry, Kate DeSear, Jason C Gallagher, Esther Golnabi, Sarah B Green, Alan E Gross, Emily L Heil, Krutika M Hornback, Keith S Kaye, Trieu-Vi Khuu, Megan Klatt, Ellen G Kline, Ryan C Kubat, Wesley D Kufel, Alexander Lepak, Conan MacDougall, Anjali Majumdar, Amy Mathers, Erin K McCreary, William R Miller, Marguerite Monogue, W Justin Moore, Shannon Olson, Jessica Oxer, Jeffrey C Pearson, Christine Pham, Paulette Pinargote, Christopher Polk, Michael J Satlin, Sarah W Satola, Sunish Shah, Pranita Tamma, Truc Cecilia Tran, David van Duin, Mollie VanNatta, Ana Vega, Venugopalan Veena, Michael P Veve, Walaiporn Wangchinda, Lucy S Witt, Janet Wu, Ryan K Shields

**Affiliations:** University of Michigan, College of Pharmacy, Ann Arbor, MI; University of Miami Miller School of Medicine, Jackson Health System, Aventura, FL; Atrium Health, Charlotte, North Carolina; Michigan Medicine, Ann Arbor, MI; Emory University Hospital, Atlanta, Georgia; Emory University, Atlanta, GA; Medical University of South Carolina, Charleston, South Carolina; University of Maryland Baltimore, Baltimore, Maryland; University of Illinois at Chicago, Chicago, Illinois; UF Health Shands Hospital, Gainesville, FL; Temple University, Philadelphia, PA; UT Southwestern, Dallas, Texas; Emory University Hospital, Atlanta, Georgia; University of Illinois, Chicago, IL; University of Maryland School of Pharmacy, Baltimore, MD; MUSC Health, Mt Pleasant, SC; Rutgers Robert Wood Johnson Medical School, New Brunswick, NJ; University of North Carolina, Chapel Hill, North Carolina; The University of Kansas Health System, KS; University of Pittsburgh, Pittsburgh, Pennsylvania; University of Kansas, Kansas City, Kansas; Binghamton University School of Pharmacy Sciences, Binghamton, NY; University of Wisconsin School of Medicine and Public Health, Madison, Wisconsin; University of California San Francisco, San Francisco, CA; Rutgers-Robert Wood Johnson Medical School, New Brunswick, New Jersey; University of Virginia, Charlottesville, VA; University of Pittsburgh Medical Center, Pittsburgh, PA; Houston Methodist Research Institute, Houston, TX; University of Texas Southwestern Medical Center, Dallas, TX; Northwestern Medicine, Chicago, IL; Sinai-Grace Hospital Detroit Medical Center, Detroit, Michigan; Weill Cornell Medicine, New York, New York; Brigham and Women's Hospital, Boston, MA; University of California, Los Angeles; David School of Medicine/University of California, Los Angeles, Los Angeles, California; Lousiana State University Health Shreveport, Bossier City, Louisiana; Atrium Health, Charlotte, North Carolina; Weill Cornell Medicine, New York, New York; Emory University School of Medicine, Division of Infectious Diseases, Atlanta, Georgia; Antibiotic Management Program, UPMC Presbyterian Hospital, Pittsburgh, PA, Pittsburgh, Pennsylvania; Johns Hopkins School of Medicine, Baltimore, MD; Houston Methodist Research Institute, Houston, TX; University of North Carolina at Chapel Hill, Chapel Hill, NC; Ochsner LSU Health Shreveport, Shreveport, Louisiana; Jackson Memorial Hospital, Miami, Florida; University of Florida, College of Pharmacy, Gainesville, Florida; Eugene Applebaum College of Pharmacy and Health Sciences, Detroit, Michigan; University of Michigan College of Pharmacy, Ann Arbor, Michigan; Emory University, Atlanta, GA; Cleveland Clinic, Cleveland, Ohio; University of Pittsburgh, Pittsburgh, Pennsylvania

## Abstract

**Background:**

CACTUS is a retrospective, matched, multicenter study comparing the efficacy of C/T and CZA for the treatment of bacteremia or pneumonia due to MDR *P. aeruginosa.* We previously demonstrated that treatment with C/T resulted in higher rates of clinical success compared to CZA. The objective of this analysis is to compare the day 30 Desirability of Outcome Ranking (DOOR) between matched patient pairs.

**Figure:**
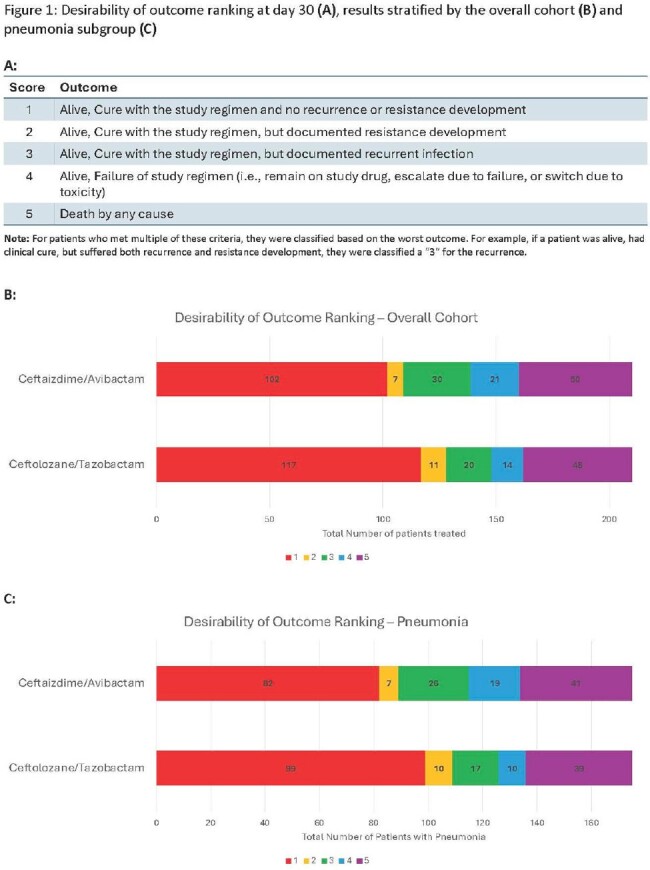

**Methods:**

C/T and CZA patients were matched 1:1 within each study site based on severity of illness, infection type, and time to treatment initiation. The DOOR scale applied is described in **Figure 1**. Each matched pair was compared for a better ranking. The frequency of an improved DOOR for C/T compared to CZA was determined for the entire cohort (n = 210 pairs) and pneumonia subgroup (n = 175 pairs) where a DOOR of 50% would represent no difference. In addition, we measured the magnitude of DOOR differences within matched pairs.

**Figure:**
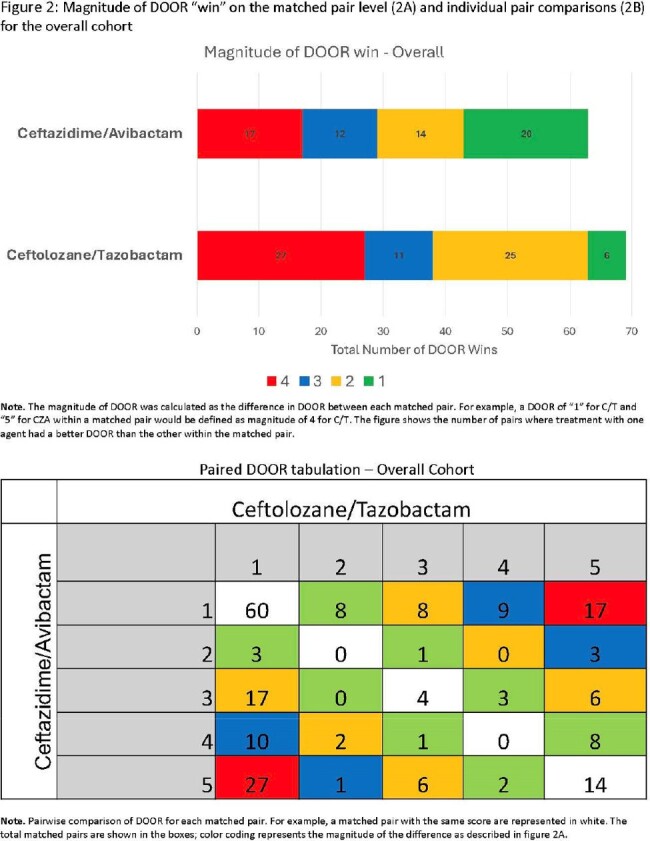

**Results:**

C/T was not associated with an improved DOOR relative to CZA in the overall cohort (53.3% (95% CI 47.2 – 59.4)) or pneumonia subgroup (54.6% (95% CI 47.9 – 61.2); **Figure 1**). Specifically, 37% of pairs had the same DOOR, 33% had a lower (better) DOOR with C/T, and 30% had a lower DOOR with CZA. **Figure 2** shows the magnitude of DOOR differences for patient pairs. The magnitude was greater for pairs when C/T was associated with a better outcome as compared to the magnitude for pairs when CZA was associated with a better outcome. This difference was more pronounced among patients with pneumonia (**Figure 3**). A summary of major comparisons is shown in **Figure 4.** For the entire cohort, an improved DOOR by ≥2 categories within matched pairs occurred more commonly with C/T vs. CZA (p=0.03). In the subgroup of patients with pneumonia, a greater proportion of pairs showed the greatest possible difference (success without complications (DOOR=1) versus death (DOOR=5)) in favor of C/T compared to pairs in favor of CZA; 14% vs. 7%; p = 0.06.

**Figure:**
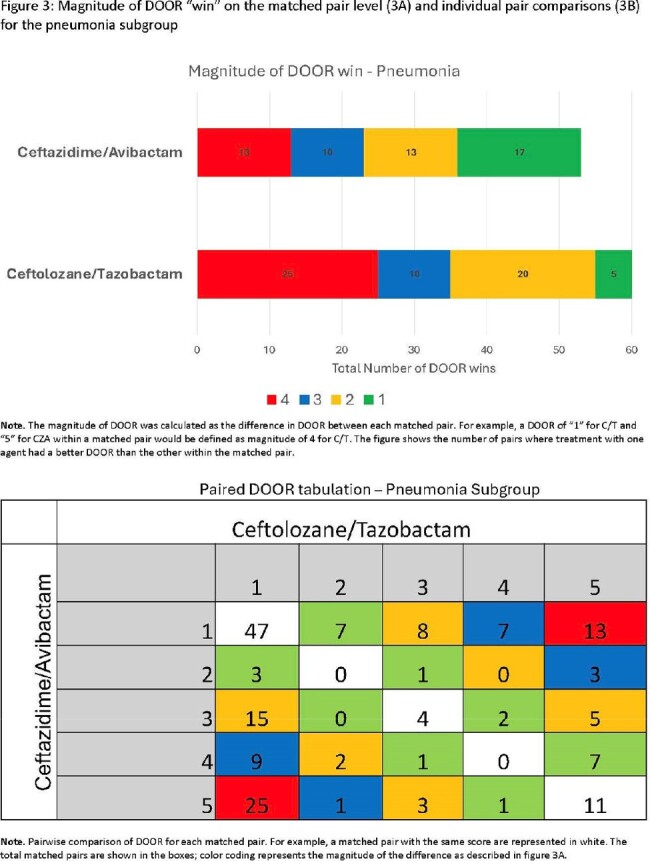

**Conclusion:**

These data demonstrate the power of matched data for analyzing DOOR endpoints. While there was no difference in the overall DOOR, the magnitude of improved outcomes consistently favored C/T over CZA and suggests clinically important differences in these agents for patients with MDR *P. aeruginosa* pneumonia or bacteremia.

**Figure:**
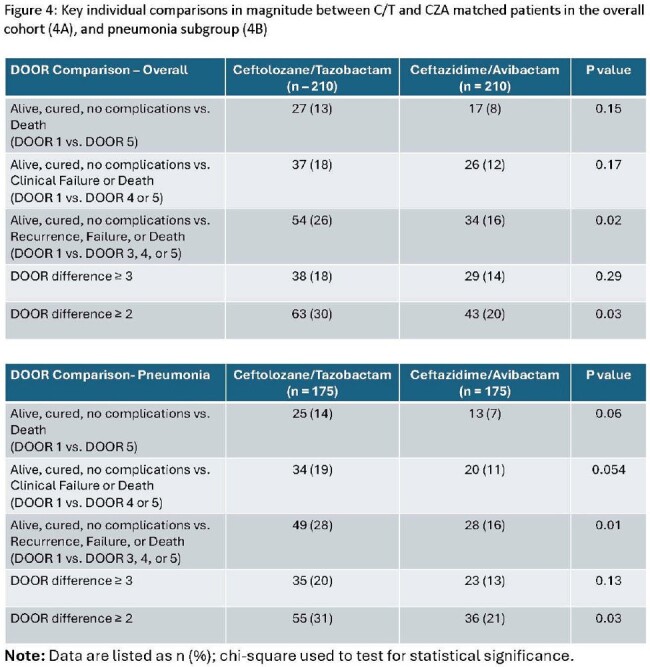

**Disclosures:**

**jason M. Pogue, PharmD**, Entasis: Advisor/Consultant|GSK: Advisor/Consultant|Melinta: Advisor/Consultant|Melinta: Grant/Research Support|Merck: Advisor/Consultant|Merck: Grant/Research Support|Shionogi: Advisor/Consultant|Shionogi: Grant/Research Support|Venatorx: Advisor/Consultant **Samuel L. Aitken, PharmD, MPH**, Basilea: Advisor/Consultant|bioMerieux: Advisor/Consultant|Melinta: Advisor/Consultant|Shionogi: Advisor/Consultant **Ahmed Babiker, MBBS**, Beckman Coulter Inc.: Advisor/Consultant **Kimberly C. Claeys, PharmD, PhD**, bioMérieux: Advisor/Consultant|bioMérieux: Honoraria **Kate DeSear, PharmD, BCIDP, AAHIVP, FIDSA**, AbbVie Inc: Advisor/Consultant|Basilea Pharmaceutica: Advisor/Consultant|GSK: Advisor/Consultant|La Jolla (Entasis): Advisor/Consultant|Melinta Therapuetics: Advisor/Consultant **Alan E. Gross, PharmD**, Becton Dickinson Co: Advisor/Consultant **Keith S. Kaye, MD, MPH**, Allecra: Advisor/Consultant|CARB-X: Advisor/Consultant|GSK: Advisor/Consultant|Merck: Advisor/Consultant|Shionogi: Advisor/Consultant|Spero: Advisor/Consultant **Wesley D. Kufel, Pharm.D., BCPS, BCIDP**, Merck & Co.: Grant/Research Support|Shionogi, Inc: Grant/Research Support **Conan MacDougall, PharmD, MAS**, Merck: Grant/Research Support **Erin K. McCreary, PharmD**, Abbvie: Advisor/Consultant|Basilea: Advisor/Consultant|Ciadara: Advisor/Consultant|Entasis: Advisor/Consultant|Ferring: Advisor/Consultant|GSK: Advisor/Consultant|GSK: Honoraria|Melinta: Advisor/Consultant|Merck: Advisor/Consultant|Pfizer: Honoraria|Shionogi: Advisor/Consultant|Shionogi: Honoraria **William R. Miller, M.D.**, Merck: Grant/Research Support|UptoDate: Royalties **Jeffrey C. Pearson, PharmD**, inflarx: Advisor/Consultant **Michael J. Satlin, MD**, AbbVie: DSMB participant|bioMerieux: Grant/Research Support|Merck: Grant/Research Support|Selux Diagnostics: Grant/Research Support|SNIPRBiome: Grant/Research Support **David van Duin, MD, PhD**, Merck: Advisor/Consultant|Merck: Grant/Research Support|Pfizer: Advisor/Consultant|Qpex: Advisor/Consultant|Roche: Advisor/Consultant|Shionogi: Advisor/Consultant|Shionogi: Grant/Research Support **Ryan K. Shields, PharmD, MS**, Allergan: Advisor/Consultant|Cidara: Advisor/Consultant|Entasis: Advisor/Consultant|GSK: Advisor/Consultant|Melinta: Advisor/Consultant|Melinta: Grant/Research Support|Menarini: Advisor/Consultant|Merck: Advisor/Consultant|Merck: Grant/Research Support|Pfizer: Advisor/Consultant|Roche: Grant/Research Support|Shionogi: Advisor/Consultant|Shionogi: Grant/Research Support|Utility: Advisor/Consultant|Venatorx: Advisor/Consultant|Venatorx: Grant/Research Support

